# Distinct Macrophage Fates after *in vitro* Infection with Different Species of *Leishmania*: Induction of Apoptosis by *Leishmania (Leishmania) amazonensis*, but Not by *Leishmania (Viannia) guyanensis*

**DOI:** 10.1371/journal.pone.0141196

**Published:** 2015-10-29

**Authors:** Jarina Pena DaMata, Bárbara Pinheiro Mendes, Kátia Maciel-Lima, Cristiane Alves Silva Menezes, Walderez Ornelas Dutra, Lirlândia Pires Sousa, Maria Fátima Horta

**Affiliations:** 1 Departamento de Bioquímica e Imunologia, Instituto de Ciências Biológicas, Universidade Federal de Minas Gerais, Belo Horizonte, MG, Brazil; 2 Departamento de Morfologia, Instituto de Ciências Biológicas, Universidade Federal de Minas Gerais, Belo Horizonte, MG, Brazil; 3 Departamento de Análises Clínicas e Toxicológicas, Faculdade de Farmácia, Universidade Federal de Minas Gerais, Belo Horizonte, MG, Brazil; Department of Medical Lab Technology, Faculty of Applied Medical Sciences, Taibah University, SAUDI ARABIA

## Abstract

*Leishmania* is an intracellular parasite in vertebrate hosts, including man. During infection, amastigotes replicate inside macrophages and are transmitted to healthy cells, leading to amplification of the infection. Although transfer of amastigotes from infected to healthy cells is a crucial step that may shape the outcome of the infection, it is not fully understood. Here we compare *L*. *amazonensis* and *L*. *guyanensis* infection in C57BL/6 and BALB/c mice and investigate the fate of macrophages when infected with these species of *Leishmania in vitro*. As previously shown, infection of mice results in distinct outcomes: *L*. *amazonensis* causes a chronic infection in both strains of mice (although milder in C57BL/6), whereas *L*. *guyanensis* does not cause them disease. *In vitro*, infection is persistent in *L*. *amazonensis*-infected macrophages whereas *L*. *guyanensis* growth is controlled by host cells from both strains of mice. We demonstrate that, *in vitro*, *L*. *amazonensis* induces apoptosis of both C57BL/6 and BALB/c macrophages, characterized by PS exposure, DNA cleavage into nucleosomal size fragments, and consequent hypodiploidy. None of these signs were seen in macrophages infected with *L*. *guyanensis*, which seem to die through necrosis, as indicated by increased PI-, but not Annexin V-, positive cells. *L*. *amazonensis*-induced macrophage apoptosis was associated to activation of caspases-3, -8 and -9 in both strains of mice. Considering these two species of *Leishmania* and strains of mice, macrophage apoptosis, induced at the initial moments of infection, correlates with chronic infection, regardless of its severity. We present evidence suggestive that macrophages phagocytize *L*. *amazonensis*-infected cells, which has not been verified so far. The ingestion of apoptotic infected macrophages by healthy macrophages could be a way of amastigote spreading, leading to the establishment of infection.

## Introduction

Leishmaniasis is a broad spectrum disease caused by over 20 different species of protozoa of the genus *Leishmania*. The disease manifests with four major outcomes, namely, localized cutaneous, diffuse cutaneous, mucocutaneous and visceral forms [1], which depend on parasite species, degree and primary site of infection, immune response and genetic predisposition of the host [2]. An estimated 12 million cases of leishmaniasis exist worldwide with 1.5–2 million new cases occurring annually, of which cutaneous leishmaniasis accounts for more than 50% [2, 3]. Over 350 million people in 88 countries live at risk of infection [1].

*Leishmania (Leishmania) amazonensis* and *L*. *(Viannia) guyanensis* are species associated with cutaneous leishmaniasis, although they differ in several aspects, including the kind of lesion and the type of immune response they cause. *L*. *amazonensis* accounts for a broad spectrum of diseases, with cases of simple cutaneous lesions, but also extending from anergic diffuse cutaneous to mucocutaneous or visceralization [4–6]. *L*. *guyanensis* causes small and numerous cutaneous ulcers, usually without any mucosal secondary involvement [7, 8]. In the murine model, it has been shown that these species also behave rather differently, producing diverse outcomes, according to the mouse strain used for infection. C57BL/6 and BALB/c mice are widely compared strains in the study of leishmaniasis due to their contrasting reactions to infection with some species of *Leishmania*. C57BL/6 mice are usually resistant to infection with *L*. *guyanensis*, but develop a chronic disease when infected with *L*. *amazonensis*, whereas BALB/c are extremely vulnerable to *L*. *amazonensis*, but like C57BL/6, totally resistant to *L*. *guyanensis* [9, 10]. In *L*. *amazonensis* infections, susceptibility in both mice strains is related with activation of both Th1 and Th2 response, with low production of IFN-γ, IL-10, IL-17 and IL-4 [11, 12]. A rapid and transient accumulation of Treg cells in the initial weeks of infection in C57BL/6, which modulates IFN-γ production helps restraining disease progression [13, 14]. Little is known about the *L*. *guyanensis* Th response of infected mice. Previous results from our group have suggested that the innate respiratory burst is involved in BALB/c mice resistance [10]. In C57BL/6 MyD88- and TLR9-dependent IL-12 production induces a protective Th1 response [15]. IL-12 was also shown to be protective in humans infected with *L*. *guyanensis*, while IL-13 favors the persistence of the infection by rendering CD4+ T cells unresponsive to IL-12 [16, 17]. T regs and IL-10 produced by T CD8+ also have a role in the pathogenesis of human *L*. *guyanensis* infection [18, 19].

Vertebrate hosts are infected when female sandflies inoculate, during blood meal, infective promastigotes into the dermis, where they are ultimately phagocytized by macrophages. Within these cells, promastigotes differentiate into replicative amastigotes, which settle inside acidic phagolysosomes. Amastigotes eventually leave infected macrophages and are taken up by neighboring healthy cells. Infected cells can also be ingested by sandflies, where amastigotes differentiate into infective promastigotes, closing the cycle [20].

The replication of amastigotes, together with their escape from macrophage killing mechanisms, and their continuous infectivity to bystander cells, amplifies the infection and, thus, the severity of the disease [21–23]. How amastigotes leave macrophages to infect neighboring cells is not yet understood. It has usually been presumed, based on static images only, that unrestricted replication of the amastigotes directly causes host cell rupture [24–27] or that amastigotes are released by exocytosis with membrane shriveling, but without cell rupture [28]. Based on our findings that *Leishmania* have a pore-forming cytolysin that we call leishporin, we have been proposing that amastigotes egress from the macrophages is mediated by pore formation on the parasitophorous vacuole and the plasma membrane [29, 30]. However, whether there is damage to both the parasitophorous vacuole and the plasma membrane and whether the macrophage dies in the process is still controversial.

Polymorphonuclear neutrophils have also been implicated in the silent transfer of intracellular parasites to macrophages. Neutrophils are short-lived cells that undergo apoptosis exposing phosphatidylserine (PS) in the outer leaflet of cell membrane, which is an ‘eat me’ signal for macrophages [31]. It has been shown that infection of neutrophils with *L*. *major* promastigotes delays, but does not prevent spontaneous granulocyte apoptosis. Therefore, neutrophils that have ingested but not killed *Leishmania* promastigotes act as safe targets for the survival of the parasites until macrophages phagocytize infected cells [32, 33].

A non-exclusive possibility would be that *Leishmania*-infected macrophages also die through apoptosis, exposing PS on their surface being phagocytized by neighboring healthy macrophages, propagating the infection. In fact, recently, Real *et al*. (2014) [34] have elegantly shown by multidimensional live cell imaging that, *in vitro*, *L*. *amazonensis* amastigotes are indeed transferred from cell to cell, apparently when the donor host macrophage delivers warning signs of imminent apoptosis. However, there are no reports on classic apoptosis of macrophages induced by the infection of *L*. *amazonensis* or *L*. *guyanensis*. Therefore, the aim of this study was to determine whether these cutaneous leishmaniasis-causing species are able to induce apoptosis in macrophages, trying to relate this process with the outcome of disease in mice. Our results show that *L*. *amazonensis* induces classic apoptosis in murine macrophages *in vitro*, from both C57BL/6 and BALB/c, showing PS exposure, classic DNA fragmentation and consequent hypodiploidy. None of these signs are seen in macrophages infected by *L*. *guyanensis*, which seem to die through necrosis. Caspases-3, -8 and -9 are activated in *L*. *amazonensis*-infected apoptotic cells from both strains of mice. Considering these two species of *Leishmania* and strains of mice, macrophage apoptosis induced at the initial moments of *in vitro* infection correlates with chronic infection, regardless of its severity.

## Material and Methods

### Animals and Ethics Statement

Male BALB/c and C57BL/6 mice, 6 to 12 weeks of age, were purchased from Centro de Bioterismo facility, Instituto de Ciências Biológicas, Universidade Federal de Minas Gerais (UFMG), Belo Horizonte, Brazil. This study was carried out in strict accordance with Brazilian laws governing animal experimentation. All procedures described here had prior approval from Animal Experimentation Ethics Committee of UFMG (Permit Number CEUA—109/2012) and all efforts were made to minimize suffering. Condition of the animals was monitored three times a week and no unintended deaths of animals occurred during this study. Euthanasia was performed by cervical dislocation by a trained person who ensured instantaneous animal death without pain or stress.

### Consumables

Equipment, consumables and software were purchased from: **Brazil**: Cripion Biotecnologia Ltda, Andradina, SP; Mitutoyo, Santo Amaro, SP; **USA**: Amersham, GE Healthcare Biosciences, Pittsburgh, PA; BD Biosciences, Franklin Lakes, NJ; Bio-Rad laboratories, Inc., Hercules, CA; Cell Signaling—Danvers, MA; Difco, Kansas City, USA; FlowJo, LLC/Tree Star, Ashland, OR; Gibco Life Technologies, Grand Island, NY; GraphPad Software, Inc., La Jolla, CA; ImageJ 1.44p software (available at http://imagej.nih.gov/ij/) National Institutes of Health, Bethesda, MD; Nalgene Nunc International, Penfield, New York; Packard, Meriden, CT; Promega Corporation, Fitchburg, WI; Santa Cruz Biotechnology, Santa Cruz, CA; Sigma-Aldrich, St. Louis, MO; Stratagene-Agilent Technologies, Santa Clara, CA; **France:** Vilmer Loumart, Marne-la-Vallée; **Germany:** Sarstedt Inc., Nümbrecht.

### Parasites

*L*. *(L*.*) amazonensis* PH8 (IFLA/BR/67/PH8) and *L*. *(V*.*) guyanensis* (MHOM/BR/75/M4147) promastigotes were axenically cultured at 24°C in Schneider medium supplemented (Sigma) with 10% heat-inactivated (hi) fetal bovine serum (FBS) (Cripion Biotecnologia Ltda). Promastigotes were harvested on the 4^th^ or 5^th^ day of culture, during the stationary phase of growth, for *in vitro* and *in vivo* infections.

### Macrophage culture and infection with promastigotes *in vitro*

Peritoneal macrophages were obtained from mice 4 days after i.p. injection of 2mL of 3% thioglycollate medium (Difco), as previously described [35]. Cells were washed with PBS, and resuspended in RPMI 1640 (Gibco) supplemented with 10% hi FBS and 50mg/mL of gentamycin. Cells were plated on 8-well chamber LabTek^®^ (Nalge Nunc International) tissue culture slides, 96-, 24-, and 6-well flat bottom plates (Nalge Nunc International), with 1 x 10^5^ cells, 5 x 10^4^, 5 x 10^5^, and1 x 10^7^, respectively, and left at 37°C in 5% CO_2_ for 2h for macrophages adherence. Adherent cells were then washed with room temperature RPMI and further incubated overnight under the same conditions.

Macrophages were infected with stationary phase promastigotes of *L*. *amazonensis* or *L*. *guyanensis* at a 10:1 parasite:macrophage ratio and incubated at 34°C at 5% CO_2_ for 4h. Cells were then washed twice to remove non-adhered or -interiorized parasites and we considered this point as the initial time of infection. Cultures of infected macrophages were incubated at same conditions for up to 4 days. Cells on the slides were fixed with methanol and stained with May-Grünwald, followed by Giemsa, method. The stained slides were used for determining the percentage of infected macrophages and the number of intracellular parasites after counting 400 or 200 macrophages, respectively, in duplicate with an optical microscope (Olympus—CX31). Infection index (percentage of infected cells x number of parasites per infected cells) was also determined. Images were obtained using QCapture Pro 6 Imaging Software (QImaging) obtained from http://www.qimaging.com/products/software/qcappro7.php.

### *In vivo* infection

Stationary phase promastigotes of *L*. *amazonensis* or *L*. *guyanensis* (1 × 10^6^) in 40 μL of PBS were inoculated subcutaneously into the right hind footpad of 6-week-old BALB/c or C57BL/6 male mice (n = 5). The evolution of the disease was monitored by weekly measuring footpad thickness with a metric caliper (Mitutoyo) and expressed as the difference in thickness between the infected and the uninfected footpad. BALB/c mice infected with *L*. *amazonensis* were sacrificed after the 10^th^ week due to the development of large necrotic lesions, while C57BL/6 infected with *L*. *amazonensis* or both strains infected with *L*. *guyanensis* were observed for 14 or 20 weeks, respectively.

### Detection of PS on infected macrophages and evaluation of cell permeability

Adhered cells, infected or not with *L*. *amazonensis* or *L*. *guyanensis* were removed with cell scrapers, washed twice with ice-cold RPMI medium and kept at 34°C with 5% CO2 in suspension in 5mL polypropylene tubes (Sarstedt) overnight for recovering [36]. Staining with Annexin V(AnnV)-FITC (BD Bioscience Pharmingen) was proceeded according to manufacturer and counterstained with PI (Sigma-Aldrich). Briefly, cells were centrifuged, washed twice with ice-cold PBS and resuspended in binding buffer (10mM HEPES/NaOH pH 7.4, 140mM NaCl and 2.5mM CaCl_2_). Cells were incubated with AnnV-FITC and PI for 15 minutes in the dark at room temperature. Fluorescence of samples was immediately measured in FACScan (Becton-Dickinson). For detection of cell permeability cells were stained as described above with PI. Macrophage population was gated by size (FSC) versus granularity (SSC), previously defined with anti-CD11b specific antibody (not shown). Gate analysis is shown in S1A Fig. Ten thousand events inside the macrophage gate were counted. Results were expressed as the percentage of AnnV positive and PI negative cells, indicating the early apoptosis events. Data analysis was performed with FlowJo (LLC/TreeStar) software.

### Hypodiploidy analysis

To evaluate nucleic acid content within macrophage nuclei, cells were lysed with hypotonic buffer containing propidium iodide (PI) and nuclear content was measured using FACScan (BD Biosciences), according to Riccardi & Nicoletti (2006) [37]. Briefly, after different periods of macrophage infection with promastigotes, supernatants were collected from plastic dishes and centrifuged for 10 minutes at 2000 rpm to recover cells that had detached from the plate. Adherent cells were treated with lysis buffer (0.1% sodium citrate, 0.1% Triton X-100, 50μg/mL PI) and the lysates were added to the previously pelleted detached cells and incubated at 4°C for overnight. Macrophage nuclei population was first gated by size (FSC) versus granularity (SSC), which was further gated to eliminate very low PI fluorescence. Gate analysis is shown in S1B and S1C Fig. Ten thousand events inside the nuclei gate were counted. Data analysis was performed with FlowJo (LLC/TreeStar) software. Results were expressed as the frequency of PI fluorescent nuclei indicating their amount of DNA.

### Analysis of DNA

1x10^7^ adhered cells infected or not with promastigotes, at different periods of infection, were collected using cell scrapers (Sarstedt) alongside eventually detached cells present in culture medium. Cells were washed twice with ice-cold PBS, lysed with lysis buffer (40 mM EDTA pH8, 50 mM Tris pH 8, 1% Triton) and incubated with RNAse (20μg/mL) (Promega) for 1h at 37°C. Proteinase K (Promega) was added to a final concentration of 100μg/mL and incubated at 56°C for at least 2h. Supernatants were treated twice with phenol: chloroform: isoamylic alcohol (25:24:1) (Sigma-Aldrich), centrifuged at 15000 rpm for 5 minutes at 4°C and finally treated with chloroform (Sigma-Aldrich) before overnight incubation at -20°C with 0.25M NaCl and twice the volume of pure ethanol (Promega). Samples were washed twice with ethanol 70% and the dried pellet was solubilized in TE (10 mM Tris-HCl, pH 8.0; 10 mM EDTA, pH 8.0). DNA samples were quantified using GeneQuant (Amersham, GE Healthcare Biosciences) and 1 μg of DNA of each sample was applied in 1.8% agarose gels. Gels were analyzed and documented with UV transilluminator (Stratagene-Agilent Technologies) and a camera (Vilmer Loumart).

### Preparation of cell extracts and Western Blotting Analysis

Macrophages in 6-well plates infected or not, were harvested with cell scrapers (Sarstedt) alongside with eventually detached cells, and washed twice with ice cold PBS. Cell extracts were obtained through cell homogenization with lysis buffer, as previously described [38] and centrifuged at 13,000 rpm, 10 minutes and 4°C. Samples were stored at -20°C until required. Protein quantification was assessed with Bradford reagent assay (Bio-Rad). Extracts (20μg) were fractionated by electrophoresis in SDS-PAGE 15% gels, transferred to nitrocellulose membranes (Bio-Rad), and membranes were blocked for 2h at 4°C with PBS containing 0.1% Tween-20 and 5% (w/v) of nonfat milk. After washing, membranes were incubated overnight at 4°C with anti-caspase-3 (#9665, Cell Signaling), at a 1:1000 dilution. Membranes were washed and incubated with a mouse anti-rabbit antibody conjugated with peroxidase (1:3000) (#7074, Cell Signaling). Finally, membranes were washed, and immunoreactive bands were visualized using ECL detection system as instructed by the manufacturer (GE Healthcare). Anti-β-actin was used as a load control at a 1:5000 dilution (A5316, Sigma), with anti-mouse secondary antibody conjugated with peroxidase at a 1:3000 dilution (sc-2005, Santa Cruz Biotechnology). Densitometry analyses were performed using ImageJ 1.44p software (National Institutes of Health), using β-actin and the band above pro-caspase-3 as load controls.

### Activated caspase-8 and caspase-9 detection

Caspase-8 and -9 were detected using Caspase-Glo ® 8 Assay and Caspase-Glo ® 9 Assay (Promega), respectively, according to manufacturer. Briefly, cells were cultured in 96-well plates and infected as described above. After determined periods of infection, prepared reagent was added to samples and gently mixed to homogenize. After 2h incubation, at room temperature, supernatants were transferred to 96-well plate appropriate for luminometer (Nalgene Nunc). Luminescence was measured in plate-reading luminometer (Packard—Luminocount). Luminescence is represented as RLU (relative light units).

### Statistical analysis

All results are presented as the mean ± SE or SD. Each two groups were analyzed by Student’s *t* test. A *P* value < 0.05 was considered significant. Calculations were performed using the Prism 5.0 software for Windows (GraphPad).

## Results

### Course of infection with *L*. *amazonensis* and *L*. *guyanensis* in C57BL/6 and BALB/c mice

**Fig 1:**
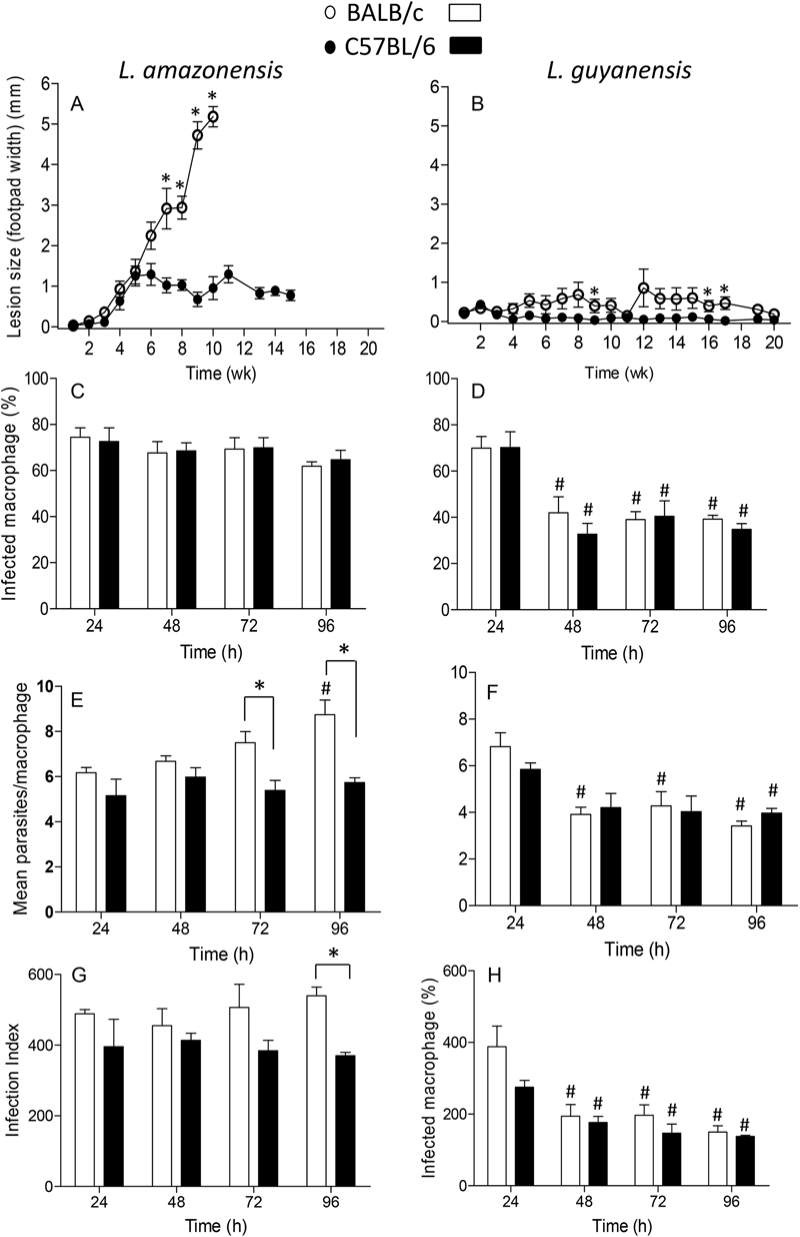
Lesion progression in mice infected with *L*. *amazonensis* or *L*. *guyanensis* and *in vitro* infection of macrophages. Groups of 5 BALB/c (open circles) or C57BL/6 (filled circles) mice were infected in one of the hind footpads with 1x10^6^ stationary phase promastigotes of *L*. *amazonensis* (A) or *L*. *guyanensis* (B). Lesion progression was assessed weekly for up to 20 weeks by measuring the hind footpads. Lesion size was expressed as the difference between infected and non-infected footpad. Each point represents mean ± SE obtained from 5 mice. Macrophages were infected with *L*. *amazonensis* (C, E, G) or *L*. *guyanensis* (D, F, H) at a 10:1 parasite:macrophage ratio and incubated for up to 4 days. Infected cells were counted and results are expressed as percentage of infected macrophages (C, D), number of parasite/cell (E, F) and infection index (G, H). Infection index was estimated as the mean percentage of infected cells x the mean number of parasites per infected cells. Bars represent mean ± SE of two to six (depending on the time point) independent experiments. Statistically significant differences between the two groups at a *P* < 0.05 are represented by (*). Statistically significant differences between values of the same group as compared to values at 24h at a *P* < 0.05 are represented by (#).

Epidemiological and experimental studies have shown that different species of *Leishmania* cause recognizably different disease outcomes. These differences are easily shown in murine models of infection, particularly in C57BL/6 and BALB/c strains of mice. Here we compare the course of infection with *L*. *amazonensis* and *L*. *guyanensis* in these two strains of mice, in which lesion growth was followed weekly (Fig 1). As observed, BALB/c mice are totally susceptible to *L*. *amazonensis* with an increasing lesion over time. Unable to control infection, mice would die if not euthanized at the 10^th^ week of infection (Fig 1A). C57BL/6 mice are much less susceptible to infection, starting with lesions comparable to those of BALB/c up to 5 weeks of infection, which, although do not progress, are persistent (Fig 1A). It is already well documented that C57BL/6 mice are not able to resolve *L*. *amazonensis*, giving rise to a chronic infection [9]. In contrast, both strains of mice completely control the lesion caused by *L*. *guyanensis*. In Fig 1B, we can see that in BALB/c, infection results in a very small footpad swelling from the 5^th^ week, which recedes to no swelling at the 20^th^ week, whereas C57BL/6 shows no lesion whatsoever during the same period.

**Fig 2:**
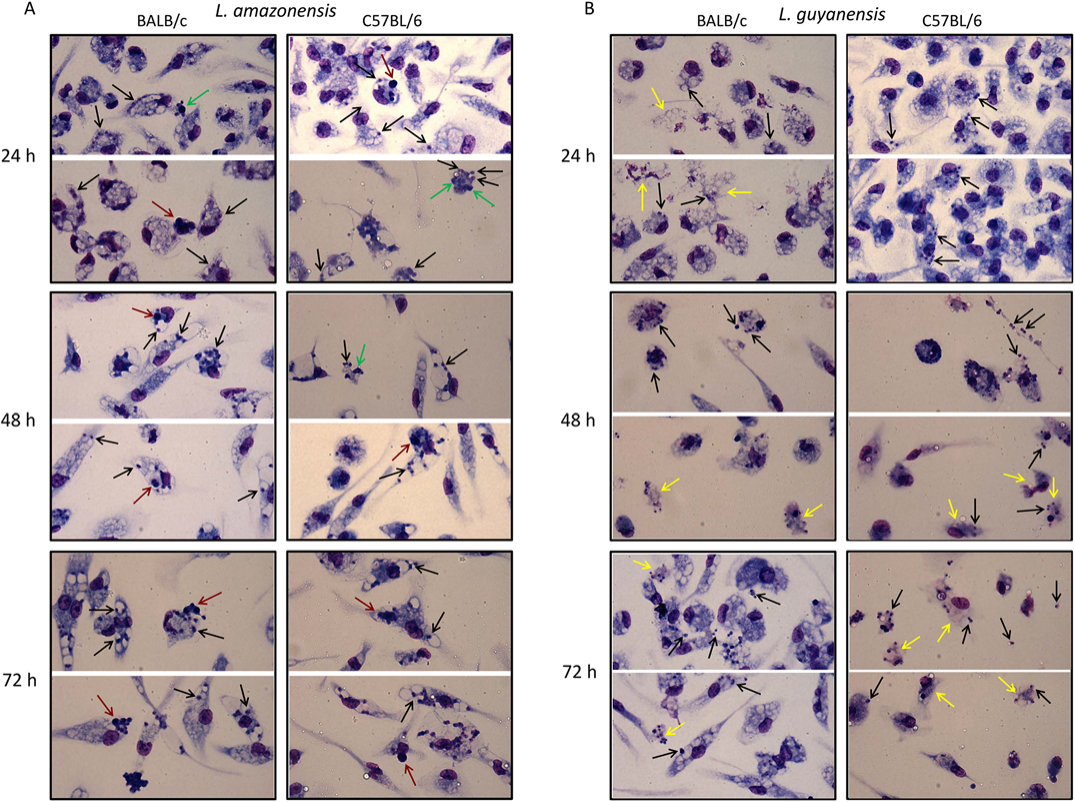
*In vitro* macrophage infection with *L*. *amazonensis* or *L*. *guyanensis*. Peritoneal macrophages of BALB/c (left panel) or C57BL/6 mice (right panel) were infected with *L*. *amazonensis* or *L*. *guyanensis*. After the indicated time points cells were stained with May-Grünwald, followed by Giemsa, method. Images were obtained using QCapture Pro 7 Imaging Software (QImaging) obtained from http://www.qimaging.com/support/softwarereleases/030107_qcappro.php. Black arrows—amastigotes; green arrows—structures reminiscent of apoptotic bodies or condensed nuclei; yellow arrows—structures reminiscent of disintegrated cells; red arrows—structures reminiscent of apoptotic cells that seem to have been phagocytized by (or are attached in) other macrophages.

*In vitro*, infection follows a pattern coherent with that of the *in vivo* infection. At the parasite:cell ratio of 10:1, almost 80% of the macrophages from both BALB/c and C57BL/6 are infected with *L*. *amazonensis* (Fig 1C) with a mean of 5–6 parasites/cell after 24 h, which increases to around 9 in 96 h in BALB/c macrophages, but remains constant in C57BL/6 cells. On the contrary, at the same conditions, not only the percentage of macrophages infected with *L*. *guyanensis* decreases from 70% to 30–40% in 48h (Fig 1D), but also the mean of parasites/cell decreases from 6–7 to 3–4 in both mice (Fig 1F), a condition that persists up to 96h. Infection index (Fig 1G and 1H) provides an approximation of the total number of parasites in the infection. Images of infected macrophages at various time points are presented on Fig 2A and 2B, where we can observe that infection with *L*. *amazonensis* is quite different from infection with *L*. *guyanensis*. *L*. *amazonensis* amastigotes (black arrows) occupy large vacuoles (Fig 2A), whereas vacuoles containing *L*. *guyanensis* amastigotes (black arrows) are not apparent (Fig 2B) in both BALB/c and C57BL/6 mice. It was frequent to observe signs of cell death such as condensed nuclei (green arrows), particularly in *L*. *amazonensis* infection, and disintegrated cells containing amastigotes (yellow arrows), particularly in *L*. *guyanensis* infection. In *L*. *amazonensis* infection apoptotic cells that seem to have been ingested by (or are attached to) other macrophages (red arrows) could also be observed. In *L*. *guyanensis* infection, at later periods, it was also frequent to observe free amastigotes among cells (black arrows). These observations prompt us to further investigate whether cell death was being caused by infection and which type of death was occurring.

### *In vitro* infection with *L*. *guyanensis*, but not with *L*. *amazonensis*, causes loss of membrane integrity in both BALB/c and C57BL/6 macrophages

**Fig 3:**
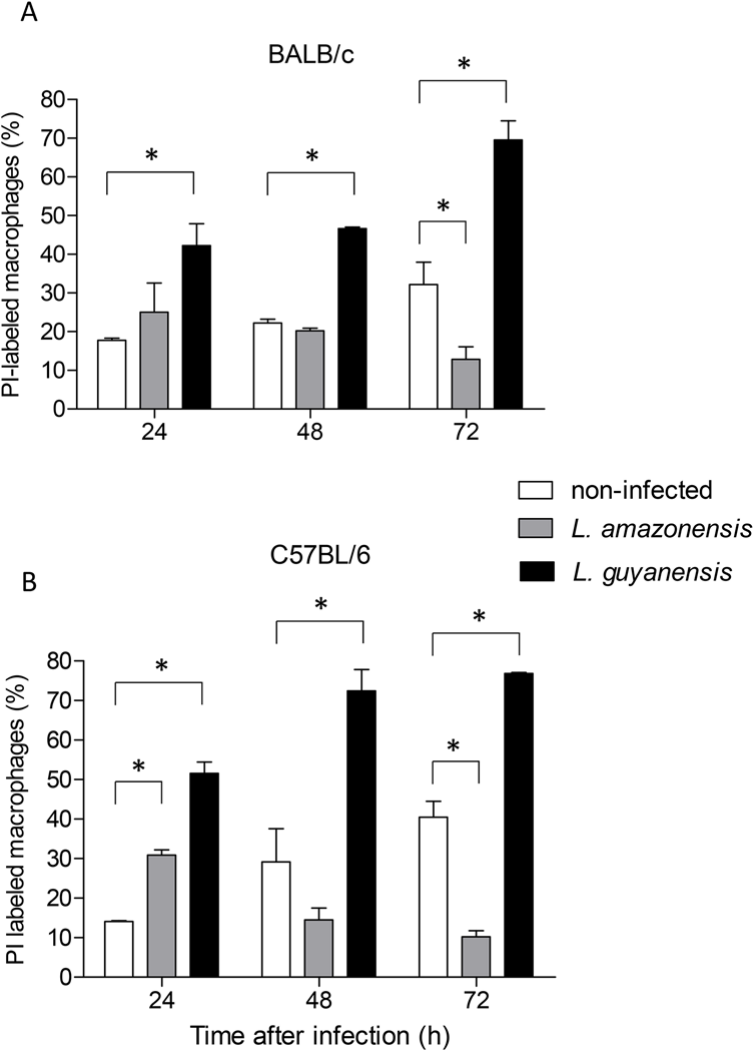
Loss of macrophage membrane integrity after *in vitro* infection with *L*. *amazonensis* or *L*. *guyanensis*. Peritoneal macrophages of BALB/c (A) or C57BL/6 (B) mice infected or not with *L*. *amazonensis* or *L*. *guyanensis* were incubated with PI and analyzed by flow cytometry at the time points indicated. Values represent percentage of PI positive cells in culture. Bars represent mean ± SE of two or three (depending on the time point) independent experiments. Statistically significant differences between the two groups indicated, at a *P* < 0.05, are represented by (*). One typical experiment is shown in dot plots after macrophage gating and analysis by FlowJo (Fig 3C). Gating strategy is shown in S1A Fig.

**Fig 4:**
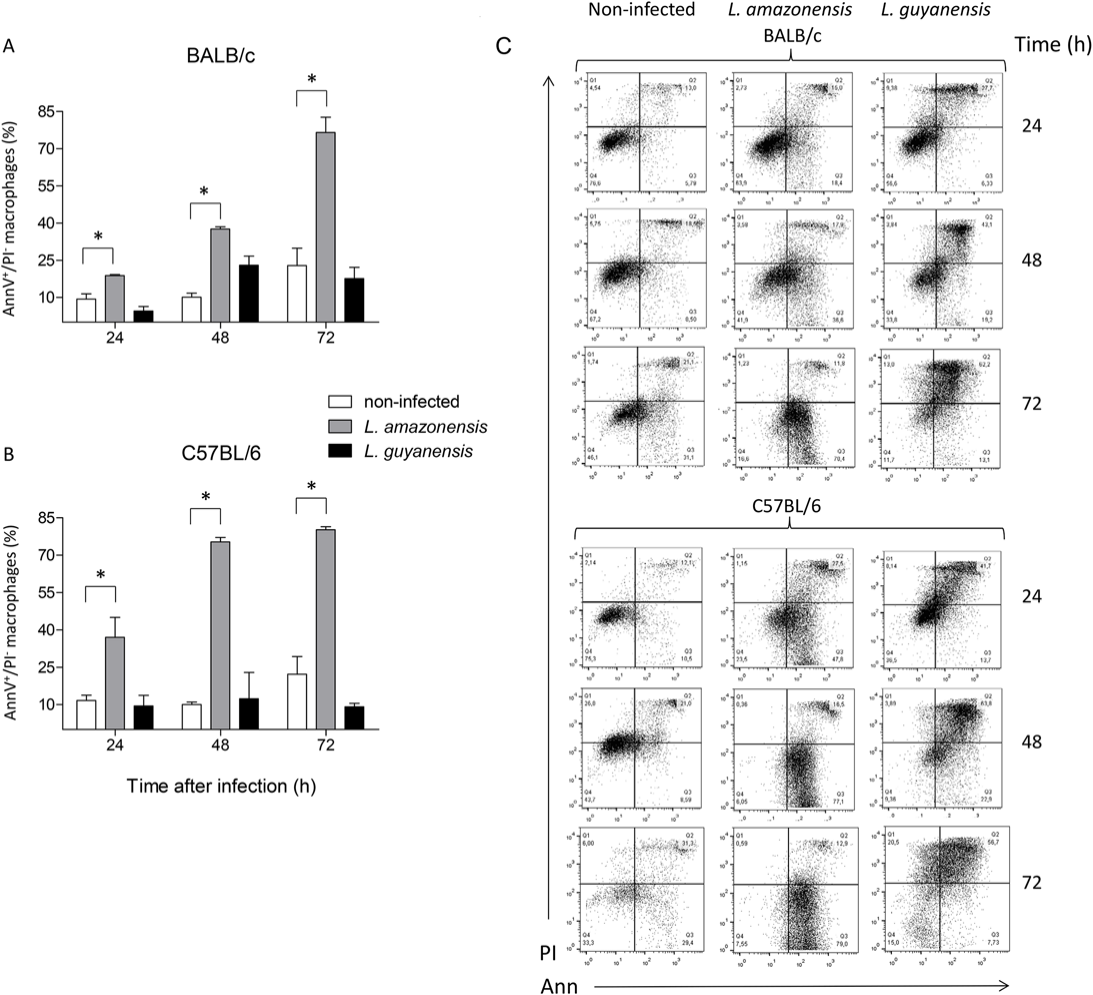
Exposure of PS by macrophages after *in vitro* infection with *L*. *amazonensis* or *L*. *guyanensis*. Peritoneal macrophages of BALB/c (A) or C57BL/6 (B) mice infected or not with *L*. *amazonensis* or *L*. *guyanensis* were labeled with AnnV-FITC and counterstained with PI. Analysis was carried out by flow cytometry and only initial moments of apoptosis (AnnV^+^/PI^-^) were considered at the indicated time points. Bars represent mean ± SE of two or three (depending on the time point) independent experiments. Statistically significant differences between the two groups indicated, at a *P* < 0.05, are represented by (*). A typical experiment is shown in dot plots after macrophage gating and analysis by FlowJo (C). Gating strategy is shown in S1A Fig.

To investigate whether these *Leishmania* species were causing death of host cells, infected macrophages were treated with PI and cell permeability was assessed by flow cytometry (Fig 3). Membrane integrity of both BALB/c (Fig 3A) and C57BL/6 (Fig 3B) macrophages infected with each of the two species shows distinct patterns, as infection progresses. *L*. *guyanensis* clearly causes loss of macrophage membrane integrity, inducing a massive macrophage death as early as 24h of infection, culminating with most cells dead at 72h. On the contrary, although *L*. *amazonensis* also may cause some loss of macrophage membrane integrity in 24h of infection, this is not observed at 48 or 72h. In fact, there are less *L*. *amazonensis*-infected cells from both strains of mice permeable to PI than uninfected cells that spontaneously die, losing membrane integrity. A typical cytometry plot can be seen in Fig 4C, in which case cells were also labeled with AnnV, as described in the following section. Simultaneously, we sought to investigate whether macrophage death caused by *L*. *guyanensis* was through apoptosis. We initially looked for the following features shown by apoptotic cells: exposure of PS, degradation and loss of DNA.

### *In vitro* infection with *L*. *amazonensis*, but not with *L*. *guyanensis*, causes macrophage exposure of PS

To detect the exposure of PS on macrophages, we incubated cells with FITC-labeled AnnV, which specifically binds to PS, and counterstained with PI (Fig 4). Unexpectedly, the massive damage of *L*. *guyanensis*-infected BALB/c or C57BL/6 macrophages was not derived from apoptotic cell death since the percentage of cells exposing PS did not exceed that of non-infected cells (the apparent difference between *L*. *guyanensis*-infected and uninfected at 48h, is not statistically significant at p < 0.05). Conversely, a substantial number of *L*. *amazonensis*-infected macrophages gradually exposed PS on their surface, indicating that these cells underwent apoptosis induced by the infection, in both BALB/c (Fig 4A) and C57BL/6 (Fig 4B). PS exposure is already evident at 24h of infection, and by 48h (C57BL/6) or 72h (BALB/c) most cells are PS positive. In this analysis, we are representing cells at early stage of death (AnnV positive/PI negative), since at the end of necrosis cells may also be labeled with AnnV. It is curious that, during the period of 48h, more than twice as many *L*. *amazonensis-*infected C57BL/6 macrophages expose PS as compared to *L*. *amazonensis-*infected BALB/c cells. Typical plots are shown in Fig 4C.

### *In vitro* infection with *L*. *amazonensis*, but not with *L*. *guyanensis*, causes loss of host cell DNA and a typical 180–200 bp-, or multiples-, ladder DNA degradation

**Fig 5:**
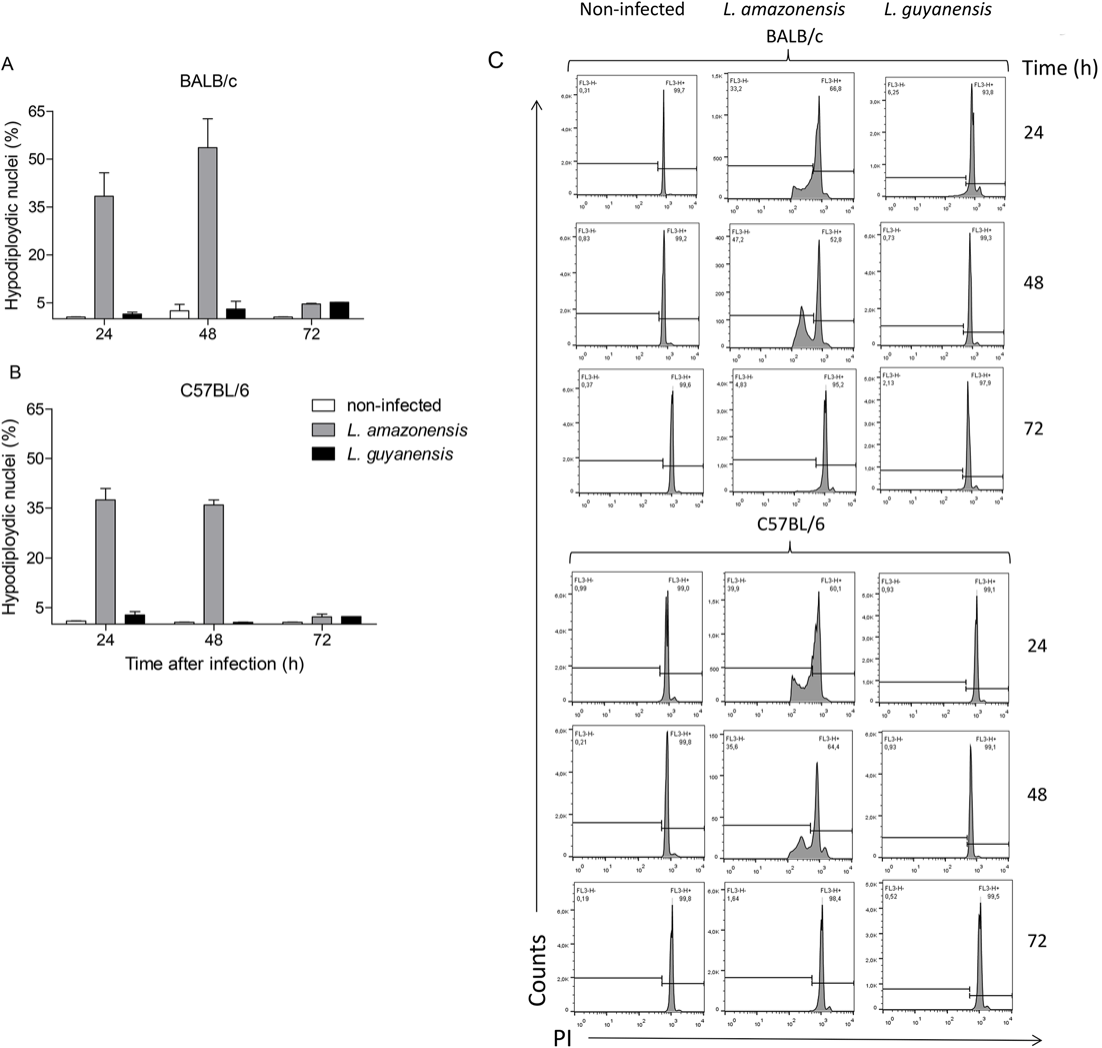
Loss of nuclear DNA by macrophages after *in vitro* infection with *L*. *amazonensis* or *L*. *guyanensis*. Peritoneal macrophages of BALB/c (A) or C57BL/6 (B) mice infected or not with *L*. *amazonensis* or *L*. *guyanensis*. After the indicated time points cells were lysed with hypotonic lysis buffer containing PI and nuclei were analyzed by flow cytometry. Bars represent mean ± SD of three independent experiments. A typical experiment is shown in histograms after macrophage nuclei gating and analysis by FlowJo. Gating strategy of cell nuclei is shown in S1B and S1C Fig.

Loss of nuclear DNA from infected macrophages was evaluated with PI staining of cell nuclei only and quantified by flow cytometry. This protocol is based on the principle that apoptotic cells have their DNA degraded by the action of endonucleases, and PI binding to DNA emits fluorescence proportional to nuclear DNA content [36]. Fig 5 shows the percentage of hypodiploidic macrophage nuclei of BALB/c (Fig 5A) and C57BL/6 (Fig 5B) mice after infection with each species of *Leishmania*. Corroborating the previous results, we could observe that *L*. *guyanensis* induces a very small loss of DNA content, whereas *L*. *amazonensis* induces a substantial DNA loss in both strains of mice, indicating that *L*. *amazonensis-*infected cells are indeed undergoing apoptosis. Typical histograms are shown in Fig 5C. To confirm that classic apoptosis was taking place, we carried out agarose gel electrophoresis to investigate how loss of DNA was occurring.

**Fig 6:**
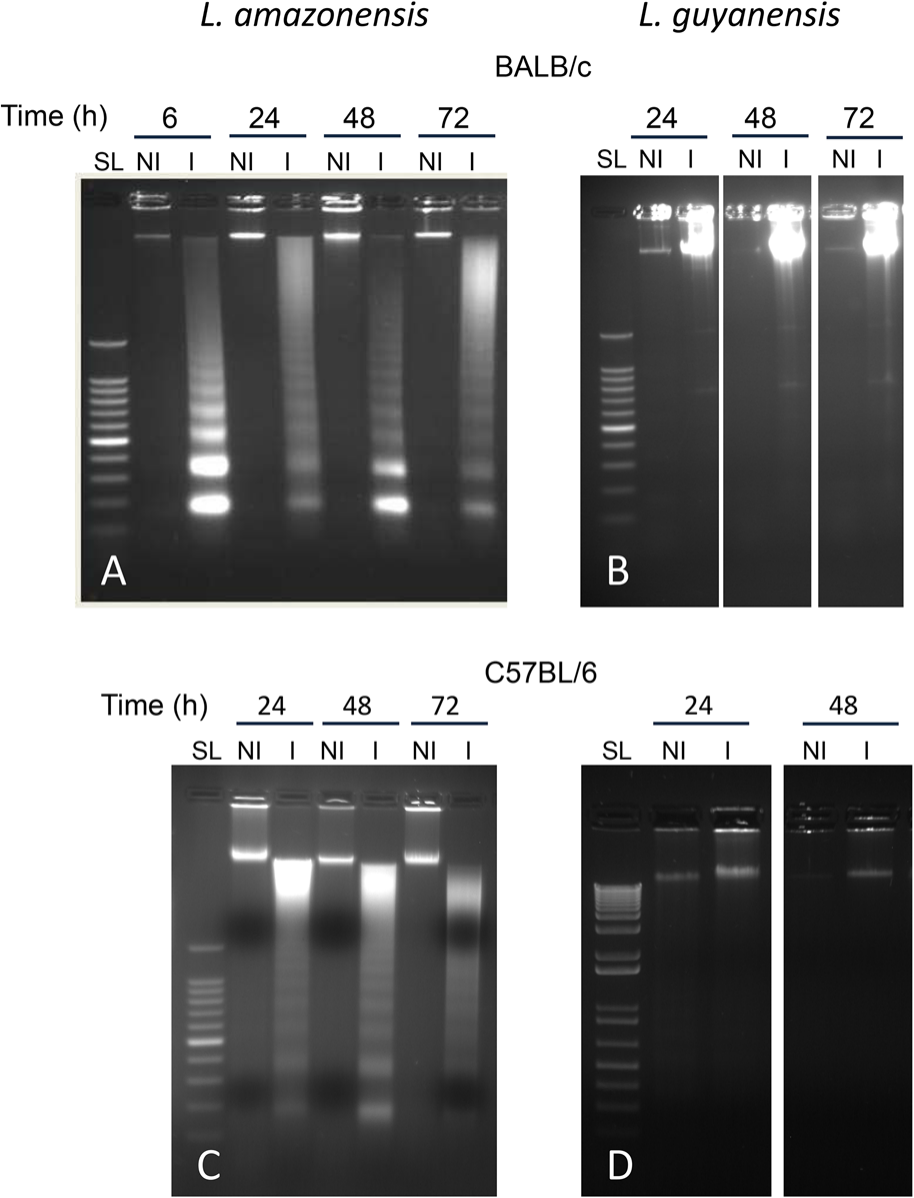
Macrophage DNA fragmentation pattern after *in vitro* infection with *L*. *amazonensis* or *L*. *guyanensis* as shown by agarose gel electrophoresis. Peritoneal macrophages of BALB/c (A and B) or C57BL/6 mice (C and D) were infected or not with *L*. *amazonensis* (A and C) or *L*. *guyanensis* (B and D). DNA was extracted after the indicated time points and submitted to electrophoresis on agarose gel at 1.8%. SL—Step Ladder of 100 bp; NI—non-infected; I—infected.

In accordance with the preceding results, we could observe the typical DNA cleavage into nucleosomal size fragments of 180–200 bp, or multiples, in macrophages infected with *L*. *amazonensis* in both BALB/c (Fig 6A) and C57BL/6 (Fig 6C). *L*. *guyanensis* does not induce DNA fragmentation in host cells from either mice strains (Fig 5B and 5D). *L*. *amazonensis*-induced DNA fragmentation can be observed as early as 6h after infection, as seen in BALB/c macrophages (Fig 6A) and is extended to up to 72h in both strains (Fig 6A and 6C). In fact, preliminary TUNEL assay results show fragmented DNA as early as 30min after parasite-host cell interaction (data not shown).

### Caspases-3, -8 and -9 are activated in *L*. *amazonensis*-infected apoptotic cells

**Fig 7:**
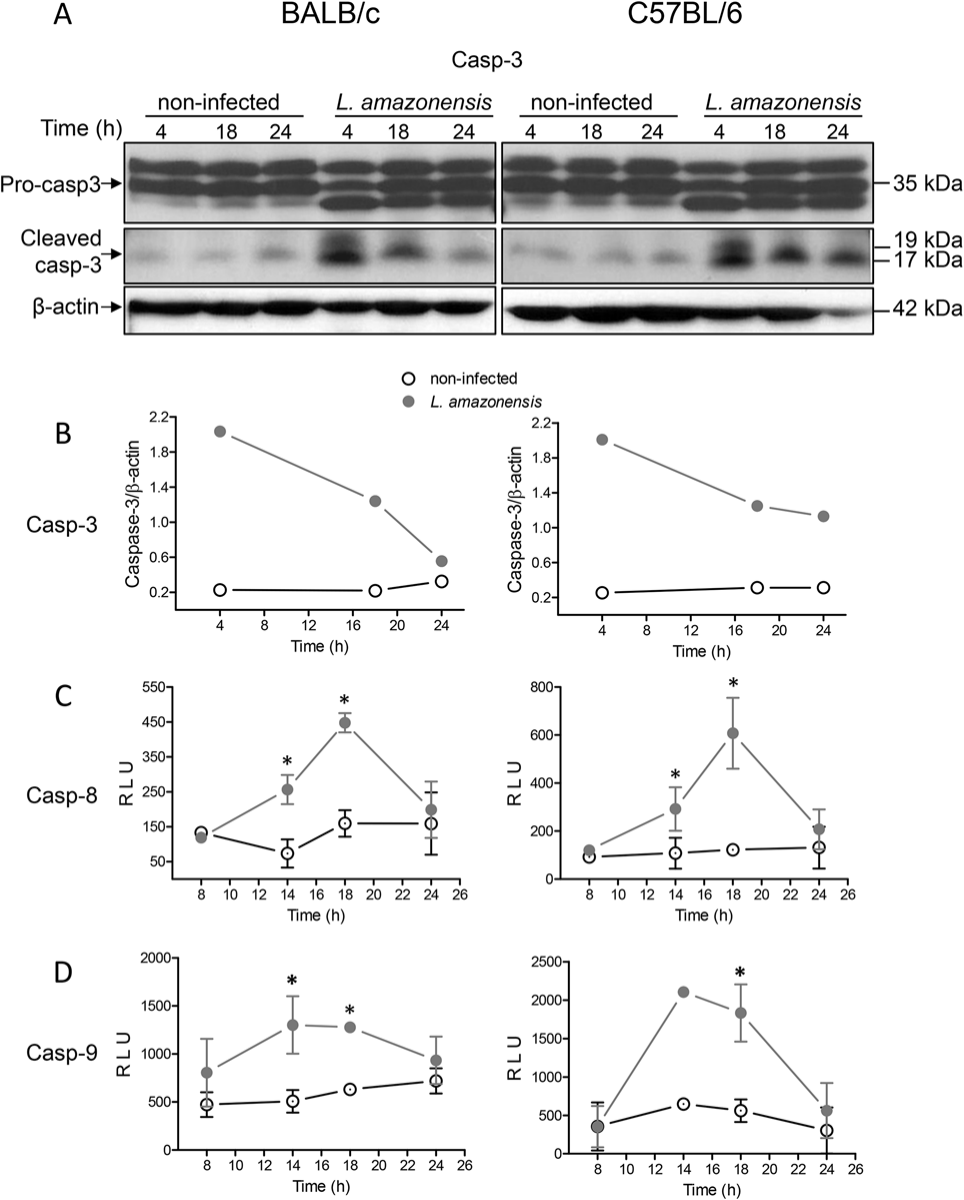
Caspase-3, -8 and -9 activation in macrophages after *in vitro* infection with *L*. *amazonensis*. Peritoneal macrophages of BALB/c (left panel) or C57BL/6 mice (right panel) were infected or not (NI) with *L*. *amazonensis*. After the indicated time points cell lysates were analysed for caspase activation. Caspase-3 activation was analyzed by Western blot (A) and quantified by densitometry using β-actin and the band above pro-caspase-3 as a load control (B). Caspase-8 and caspase-9 activation was analyzed by CaspaseGlo8 (C) or CaspaseGlo9 (D), both detected by luminescence. For caspases 8 and 9, values represent relative light units (RLU) ± SE from two, three or four (depending on the time point) independent experiments, except for time 14h for caspase-9 in C57BL/6 macrophages a value obtained from one experiment. Statistically significant differences between the two groups indicated, at a *P* < 0.05, are represented by (*).

The DNA fragmentation observed in *L*. *amazonensis*-infected macrophages suggested that CAD (caspase-activated DNAse) was activated by caspase-3. To verify whether caspase-3 was activated, protein extracts of *L*. *amazonensis*-infected macrophages from both BALB/c and C57BL/6 were analyzed by Western blot, using an anti-caspase-3 antibody, which detects endogenous levels of full-length pro-caspase-3 (35 kDa) and large fragments (17 and 19 kDa) of active caspase-3 resulting from cleavage at aspartic acid 175 (Fig 7A). Caspase-3 activation is clear in infected macrophages, as observed by the appearance of the fragments of the expected size that reacted with the specific antibody. Visualization (Fig 7A) and quantification (Fig 7B) of the bands showed that activation occurs at as early as 4h and remains activated for at least 18 or 24h post-infection in BALB/c and C57BL/6 macrophages, respectively. We also measured the activation of the upstream caspases-8 (Fig 7C) and -9 (Fig 7D) and observed that both are also activated in *L*. *amazonensis*-infected macrophages. We did not observe activation of caspases 8 and 9 up to 8h of infection. For the periods evaluated, activation peaks at 18 or 14–18h post-infection for caspase-8 and caspase-9, respectively. Infection-induced caspase-8 activation also occurred with strain BA199 of *L*. *amazonensis*, but was absent with strain M1176 of *L*. *guyanensis*, corroborating the idea that induction or non-induction of apoptosis are features associated with the species (S2 Fig).

## Discussion

Despite the amount of data on host cell-*Leishmania* interactions, the fate of infected macrophages persists as a poorly explained event, and how amastigotes spread to healthy macrophages, amplifying the infection, is still not fully understood. Transfer of amastigotes from infected to healthy cells could occur through 1) release of parasites due to cell death with rupture, 2) phagocytosis of apoptosis-dying infected cells by healthy cells without cell rupture, 3) exocytosis or extrusion of parasites inside membranous host cells structures without cell death, and/or 4) extrusion of parasites from dying cells.

Cell death/rupture passively caused by amastigote overpopulation has been the customary assumption for release of parasites [24–27]. Lately, we have proposed that a parasite-derived pore-forming cytolysin may actively damage macrophage membranes [29, 30]. It has also been proposed that cells release amastigotes through exocytosis without bursting [28]. These assumptions, however, remain to be confirmed. On the other hand, phagocytosis of *L*. *major*-infected apoptotic neutrophils by macrophages was proposed [32] and demonstrated [33] by Laskay’s group. Dubbed as the Trojan horse strategy, it is also used by *L*. *donovani* [39]. Macrophage-macrophage phagocytosis of infected cells had not been verified so far. Recently, however, it has been shown by live cell imaging that *L*. *amazonensis* amastigotes can be transferred from infected to healthy macrophages by extrusion of LAMP-rich structures containing the parasite [34]. This process was observed only after 15 days of infection *in vitro*, suggesting that it is triggered by dying host cells. In fact, although apoptosis had not been demonstrated in host cells, some apoptotic-like features, such as the extrusion of parasites trapped within zeiotic structures and the increase of anti-apoptotic genes, were shown [34].

Here, we present evidence that *L*. *amazonensis*, but not *L*. *guyanensis*, induces classic apoptosis on infected macrophages from both BALB/c and C57BL/6 mice, evidence suggestive that macrophages phagocytize *L*. *amazonensis*-infected cells, which had not been verified so far, and propose that apoptosis causes a macrophage-macrophage phagocytosis, accelerating spreading of amastigotes and contributing to more pathogenic outcomes. Apoptotic signs included PS exposure (Fig 4) and loss of DNA (Fig 5), cleaved into nucleosome-sized fragments (Fig 6). DNA fragmentation could be observed as early as 6 h (or even earlier) being present during the whole period analyzed (Fig 6). Although cells exposing PS were observed 1 day post infection, their number increasing with time (Fig 4), it probably started much earlier since PS exposure is an earlier step in apoptosis than DNA degradation. It was puzzling that, after high levels of DNA degradation up to 48 h (Figs 6 and 4), hypodiploid cells were almost absent at 72 h (Fig 5) even though PS exposure could still be observed (Fig 4). One explanation for this apparent contradiction is that at 72 h, as nuclei are mostly degraded, fewer hypodiploid nuclei are size-gated and small apoptotic bodies are excluded (Fig 2). As PS exposure is measured in whole cells, size-gating might not exclude positive fragments of cells (membrane-surrounded apoptotic bodies). In fact, apoptotic bodies, free or surrounded by membrane, could be observed at the microscope (Fig 2). Supporting this is the fact that we still see DNA fragmentation at 72 h (Fig 6). *L*. *guyanensis*-infected cells seem to die through necrosis, as evidenced by the high number of PI-permeable cells (Fig 3) and observation of ruptured cells without nuclear lesion (Fig 2).

Although *L*. *amazonensis*-infected cells were dying through apoptosis, we only observed a small loss of host cell membrane integrity at 24h of infection (Fig 2). At 48 or 72h, the percentage of PI-permeable cells was actually lower than that of the non-infected cells (Fig 3). These results suggested that cells were probably phagocytized by other macrophages before loss of membrane integrity. Indeed, apoptotic nuclei were often observed inside macrophages (Fig 2), demonstrating that macrophage-macrophage phagocytosis actually occurs. This contrasted with *L*. *guyanensis*-infected cells, which had not exposed PS on their outer membrane (Fig 4) and almost all infected cells had lost their membrane integrity at 72 h (Fig 3). Moreover, we did not observe apoptotic nuclei inside macrophages in *L*. *guyanensis* infections (Fig 2). These results also support the idea that macrophage-macrophage phagocytosis occurs only in PS-exposing infected host cells. Our results showing apoptosis in *L*. *amazonensis*-infected macrophages are in accordance with Probst et al. (2012) [40], who showed that many genes involved in apoptosis are up-regulated in host cell in response to *L*. *amazonensis* infection.

The uptake of infected PS-exposing cells by healthy macrophages could be one reason why *L*. *amazonensis*-infected macrophages maintain the *in vitro* infection for up to 96h, while *L*. *guyanensis*-infected cells decrease the degree of infection and actually kill amastigotes inside the cells [10] (and Fig 2). This mechanism had already been suggested by Getti *et al*. (2008) [41], who showed that *in vitro* infection with *L*. *aethiopica*, *L*. *tropica* and *L*. *major* induces apoptosis in human macrophages, as shown by exposure of PS, mitochondrial permeabilization and caspase activation. Apoptosis has also been described *in vitro* in mouse macrophages [42] and *in vivo* in dog macrophages [43] and mice (de Castro W and Vieira, LQ—personal communication) infected with *L*. *chagasi* (= *L*. *infantum*). All species of *Leishmania*, from both New (*L*. *amazonensis*, *L*. *chagasi*) and Old World (*L*. *aethiopica*, *L*. *tropica* and *L*. *major*), shown to cause apoptosis in host cells belong to the subgenus *Leishmania*, whereas *L*. *guyanensis*, which does not cause apoptosis in host cells, belongs to the subgenus *Viannia*. We showed here that, like the strains of the *Leishmania* species used throughout this study, the strain BA199 of *L*. *amazonensis* also activates caspase-8 whereas the strain M1176 of *L*. *guyanensis* does not, suggesting that these features are species specific. It is worth mentioning that the *L*. *guyanensis* strain used in this work contains the *Leishmania RNA virus*–1 [44] and a possibility exists that it may have a role in the induction of macrophage necrosis. Other species of the subgenus *Leishmania* and *Viannia* should be investigated in their capacity to induce apoptosis to verify whether this ability is a subgenus-specific feature. Another reason why macrophages infected with *L*. *amazonensis*, but not *L*. *guyanensis*, maintain infection could be the uptake by healthy cells of *L*. *amazonensis*-containing macrophages extrusions, as demonstrated by Real et al. (2014) [34]. Our results also corroborate their findings on the increase of the anti-apoptotic genes Bcl-2 and IGF-1 in *L*. *amazonensis*-infected macrophages and strengthen their assumption of the importance of macrophage apoptosis for parasite spreading. Indeed, Getti *et al*. (2008) [41] showed that macrophage apoptosis intensifies *Leishmania* infection.

It is curious that the speed of PS exposure in *L*. *amazonensis-*infected macrophages is higher in C57BL/6 than in BALB/c (Fig 4), which seems opposed to the reasoning that apoptosis contributes to more pathogenic outcomes. However, other signs of apoptosis do not considerably differ and, in 72h, PS exposure is already comparable (Figs 4–6). It is thus possible that the kinetics of PS appearance *in vivo* is not a preponderant factor and one-day delay does not affect the degree of susceptibility. One might also think that early inflammation may contribute to more resistant phenotypes. Besides, other factors, such as the later T cell response, widely known to be a key component to the infection outcome, must act to confer resistance or susceptibility. Our hypothesis is that early apoptosis/necrosis could intensify/hinder parasite spreading.

The assumption that CAD had been activated by caspase-3, as suggested by the typical nucleosome-sized DNA fragmentation in *L*. *amazonensis*-infected macrophages (Fig 6), was indeed confirmed in both BALB/c and C57BL/6 mice (Fig 7A and 7B). Activation of caspase-3 can be achieved by either caspase-8 or -9 [45], both of which had also been activated in *L*. *amazonensis*-infected macrophages from both strains of mice (Fig 7C and 7D). It is known that caspase-8 can be activated by stimulation of TLR3 or TLR4 [46] and that caspase-9 can be activated by caspase-8 [47, 48]. Since *Leishmania* proteoglycolipids [49] and glycoinositol phospholipids [50] are stimulators of macrophage TLR4, and that *L*. *mexicana* activates macrophages via TLR4 [51], it is reasonable to hypothesize that a TLR4/caspase-8/caspase-9 pathway takes place in *L*. *amazonensis*-induced macrophage apoptosis. We can rule out that phagocytosis is sufficient to induce apoptosis, since macrophages that have phagocytized *L*. *guyanensis* or heat-killed *L*. *amazonensis* (preliminary results) do not undergo apoptosis.

Activation of caspase-3 and DNA fragmentation are observed in 4 and 6h after infection, respectively, but it is probable that active caspase-3, and fragmented DNA, appears earlier. Indicative of this is the fact that an already large amount of active caspase-3 (Fig 7A) and fragmented DNA (Fig 6A) is observed at the first points measured. Moreover, preliminary TUNEL assay results have revealed a small percentage of cells with DNA fragmented in 30min (data not shown). The kinetics of caspase-3 activation and of DNA fragmentation and loss are consistent, but we could not observe activation of caspase-8 and -9 before 8h of infection (Fig 7C and 7D). Although the canonic hierarchy of caspase activation is caspase-8/9 activating caspase-3, it has been recently shown that caspase-3 feeds back on caspase-8 by cleaving p43 form into active p18 species [52]. It is thus possible that initial activation of caspase-3 requires small amounts of active caspase-8, undetected by luminescence, which become measurable after feedback activation by caspase-3. It is also possible that the sensitivity of the caspases-8/9 detection kits is lower than that of Western blotting.

Apoptosis is an event commonly present in modulation of inflammation, and can be involved in lesion progression/regression or in mechanisms of susceptibility/resistance in many infections. Previous studies have shown that apoptotic promastigotes of both *L*. *major* [53] and *L*. *amazonensis* [54] induces TGF-β [53, 54] and IL-10 expression [55], which facilitate leishmanial growth [53, 56, 57]. Likewise, it has been shown that phagocytosis of apoptotic neutrophils by *L*. *major*-infected BALB/c mice [58] or *L*. *amazonensis*-infected human [59] macrophages leads to an increase in parasite burden. *In vivo*, apoptotic neutrophils amplified *L*. *major* replication in BALB/c [58]. However, depending on the environment where infection takes place, engulfment of apoptotic cells can also be pro-inflammatory. In *L*. *major* infection of resistant C57BL/6 mice, apoptotic neutrophils help kill intracellular parasites by macrophages *in vitro* and prevented parasite growth *in vivo* [58]. It is possible that *L*. *amazonensis*-infected macrophages have a similar effect and, ultimately, also contribute to the outcome of infection. On the other hand, uptake of necrotic neutrophils by *L*. *amazonensis*-infected human cells induces killing of parasites [59], which is dependent on neutrophil elastase and TNF-α and mediated by reactive oxygen species. This is also in consonance with the present study, in which *L*. *guyanensis*, whose infection is controlled (Fig 1), induces necrosis in macrophages, as well as with our previous studies showing that *L*. *guyanensis* is eliminated by BALB macrophage reactive oxygen species, also through apoptosis [10]. It is possible that uptake of necrotic macrophages causes the same effect as of necrotic neutrophils.

Along the line of inflammation, because caspase-8 activated by stimulation of TLR3 or TLR4 mediates the processing of pro-IL-1β [46], it is also tempting to speculate that a TLR4/caspase-8/IL-1β pathway is activated during infection, activating caspase-3 and releasing IL-1β, which, although can produce acute inflammation, can also contribute to the non-resolution of inflammation [60]. Conceivably, this could also be one reason why *L*. *guyanensis*, which seems to induce macrophage necrosis, an inflammation-inducing event, does not cause disease in BALB/c or C57BL/6 mice, and causes in humans a more restrained illness than *L*. *amazonensis*.

While *L*. *amazonensis*, which induces apoptosis in BALB/c and C57BL/6 mice, causes disease (Fig 1A) and a persistent *in vitro* macrophage infection in both strains (Fig 1C and 1D), *L*. *guyanensis*, which does not induce apoptosis, is basically harmless to both strains (Fig 1B) and *in vitro* macrophage infection is reduced (Fig 1E and 1F). Moreover, considering that PS exposing-macrophages cause the same effect as apoptotic neutrophils [32, 33, 58, 59] or promastigotes [53, 54], in BALB/c or C57BL/6, it can be expected that the different outcomes in BALB/c and C57BL/6 (Fig 1A) may also be influenced by *L*. *amazonensis*-induced macrophage apoptosis. These correlations reinforce the current thought that apoptosis interferes with the outcome of the infection and depends on the environment where the infection takes place.

## Supporting Information

S1 Fig Gating strategy for flow cytometry. Macrophage population was gated by size (FSC) versus granularity (SSC) (A), previously defined with anti-CD11b specific antibody (not shown). Macrophage nuclei population was first gated by size (FSC) versus granularity (SSC) (B), excluding parasite nuclei. The gate excluding smaller events was chosen after running controls with only parasites nuclei. Size gate was further gated to eliminate very low PI fluorescence (G2) (C). These strategies were used in experiments described in Figs 2, 3 and 4.
(TIF)

S2 Fig Caspase-3, -8 and -9 activation in macrophages after *in vitro* infection with 2 strains of *L*. *amazonensis* and *L*. *guyanensis*. Peritoneal macrophages of BALB/c (left panel) or C57BL/6 mice (right panel) were infected or not (NI) with two strains of *L*. *amazonensis* (PH8 and BA199). After the indicated time points cell lysates were analysed for caspase-8 activation using CaspaseGlo8 detected by luminescence. Values represent mean fold increase in caspase-8 activation, as compared with uninfected controls, measured in relative light units from 2 (depending on the time point) independent experiments, except for strain M1176 of *L*. *guyanensis*, a preliminary result obtained from one experiment.
(TIF)
