# Supplementary figures and images for: Distinct Macrophage Fates after in vitro Infection with Different Species of Leishmania: Induction of Apoptosis by Leishmania (Leishmania) amazonensis, but Not by Leishmania (Viannia) guyanensis
Source: PLoS One. 2015 Oct 29;10(10):e0141196. doi: 10.1371/journal.pone.0141196 (PMC4626090; doi:10.1371/journal.pone.0141196)

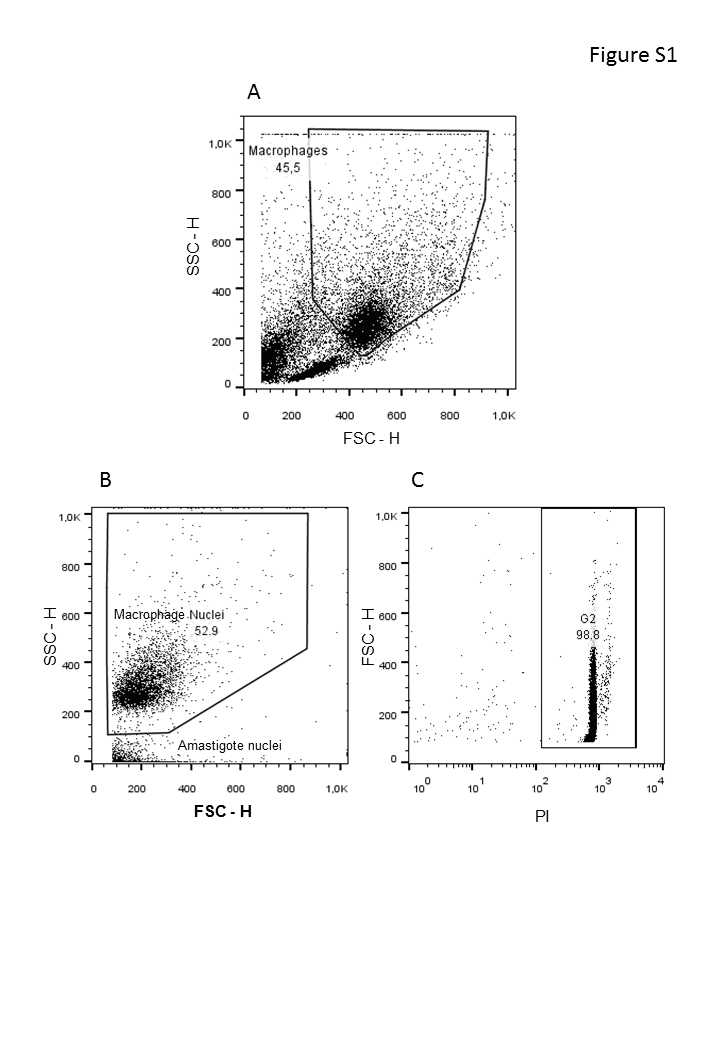

Supplement: S1 Fig — Macrophage population was gated by size (FSC) versus granularity (SSC) (A), previously defined with anti-CD11b specific antibody (not shown). Macrophage nuclei population was first gated by size (FSC) versus granularity (SSC) (B), excluding parasite nuclei. The gate excluding smaller events was chosen after running controls with only parasites nuclei. Size gate was further gated to eliminate very low PI fluorescence (G2) (C). These strategies were used in experiments described in Figs 2, 3 and 4. (TIF) [file pone.0141196.s001.TIF]

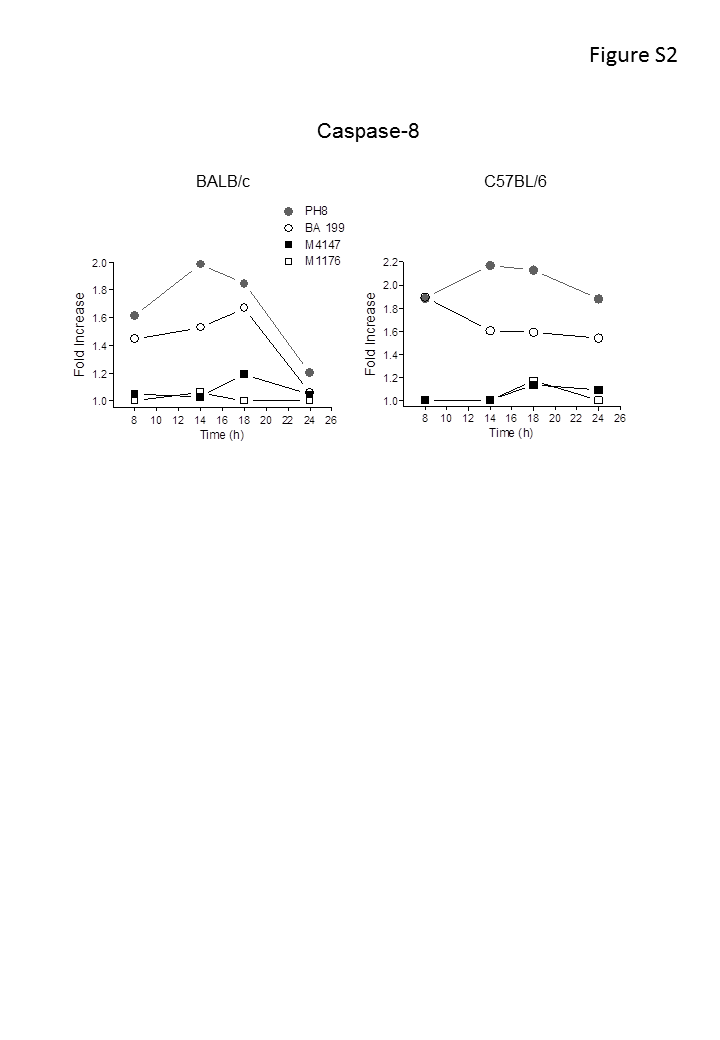

Supplement: S2 Fig — Peritoneal macrophages of BALB/c (left panel) or C57BL/6 mice (right panel) were infected or not (NI) with two strains of L. amazonensis (PH8 and BA199). After the indicated time points cell lysates were analysed for caspase-8 activation using CaspaseGlo8 detected by luminescence. Values represent mean fold increase in caspase-8 activation, as compared with uninfected controls, measured in relative light units from 2 (depending on the time point) independent experiments, except for strain M1176 of L. guyanensis, a preliminary result obtained from one experiment. (TIF) [file pone.0141196.s002.TIF]
